# Overcoming the Challenges in Implementing Type 2 Diabetes Mellitus Prevention Programs Can Decrease the Burden on Healthcare Costs in the United States

**DOI:** 10.1155/2017/2615681

**Published:** 2017-08-14

**Authors:** Kritika Subramanian, Inuka Midha, Vijaya Chellapilla

**Affiliations:** ^1^School of Medicine, University Centre, St. George's University, True Blue, Grenada; ^2^Midha Medical Clinic, 2680 Lawrenceville Hwy, Decatur, GA 30033, USA

## Abstract

Theoretically, identifying prediabetics would reduce the diabetic burden on the American healthcare system. As we expect the prevalence rate of prediabetes to continue increasing, we wonder if there is a better way of managing prediabetics and reducing the economic cost on the healthcare system. To do so, understanding the demographics and behavioral factors of known prediabetics was important. For this purpose, responses of prediabetic/borderline diabetes patients from the most recent publicly available 2015 Behavioral Risk Factor Surveillance System (BRFSS) survey were analyzed. The findings showed that there was a correlation between household income, geographic residence in the US, and risk for developing diabetes mellitus type 2, aside from the accepted risk factors such as high BMI. In conclusion, implementation of the National Diabetes Prevention Program is a rational way of reducing the burden of DM on the healthcare system both economically and by prevalence. However, difficulties arise in ensuring patient compliance to the program and providing access to all regions and communities of the United States. Technology incorporation in the NDPP program would maintain a low-cost implementation by the healthcare system, be affordable and accessible for all participants, and decrease economic burden attributed to diabetes mellitus.

## 1. Introduction

In 2016, the CDC reported that 29 million Americans were diabetic, and three times more were prediabetic [1]. Although prior studies have shown that identifying prediabetics did not prevent them from developing diabetes, medical initiatives such as behavioral modifications and medication delayed diabetic onset [2]. Individuals at risk of developing diabetes included those who were overweight, above 45 years of age, had a family history of diabetes mellitus (DM), were not physically active, and/or had gestational diabetes [1].

Recently, longitudinal studies showed an increased trend in the economic burden caused by diabetes. If a patient was 50 years old when diagnosed, the lifetime medical costs of treating DM was $135,000 undiscounted. If diagnosed ten years earlier, the cost in a lifetime treatment increased by 150% [3]. In 2012, the health burden attributed to DM was $245 billion, 43% of which was inpatient care costs. Diagnosed patients in 2012 spent approximately on average $8000 per year on medical expenses due to their diabetes. Despite the high costs and expenditures, quality of life for diabetic patients did not necessarily improve. With the aging population, there is an increase in the prevalence of diabetes and an expected increase in the attributed economic burden [4].

Theoretically, identifying prediabetics would reduce the diabetic burden on the healthcare system. Screening for prediabetes regularly begins at the age of 45 in low-risk individuals. If the initial results are normal, the subsequent screening would be conducted 3 years later. If the individual was found to be prediabetic, then annual monitoring for diabetes was recommended [5]. Screening for DM and pre-DM unfortunately was not cost effective in the majority of cases [5]. However, an exceptional study from 2010 conducted in Atlanta showed that early screening through the glucose challenge tests helped reduce overall costs for the healthcare system [6].

In the US, comprehensive prediabetic management included lifestyle interventions of diet and exercise. If the at-risk patient was less than 60 years of age, the diabetic drug metformin was prescribed [7]. Borderline DM patients were at risk for microvascular complications including nephropathies and neuropathies [5]. The cost of a prediabetic patient would therefore include physician visits, and possible fees for medications, blood glucose self-monitoring equipment, nutritionist services, monitoring and treating for microvascular complications, and weight loss consultations. In 2012, the healthcare burden in the United States for prediabetes was $44 billion [8]. As we expect the prevalence rate of prediabetes to continue increasing, we wonder if there is a better way of managing prediabetics and reducing the economic cost on the healthcare system. To do so, understanding the demographics and behavioral factors of known prediabetics was important.

## 2. Methods

Responses of prediabetic/borderline diabetes patients from the most recent publicly available 2015 Behavioral Risk Factor Surveillance System (BRFSS) survey were analyzed. The statistics and analysis were conducted using R software. Graphs were made using the ggplot2 package [9]. Chi-square calculations were also computed on base R, with expected values having uniform distribution. The BFRSS survey divided gestational diabetes and prediabetes/borderline as two separate categories. Because gestational diabetes was also a risk factor for developing Type 2 DM later in life, individuals who answered positively for having gestational diabetes were grouped together with prediabetes/borderline individuals (*n* = 11,298). Variables included in the analysis were national distribution, BMI, exercise, household income, and enrollment in a healthcare plan. National distribution of prediabetic individuals was determined by grouping the states of the survey respondents into 9 regions as defined by the US Census Bureau. Responses from surveyed individuals in Puerto Rico and Guam were not included because they did not fall into one of the nine defined regions.

## 3. Results

**Figure 1:**
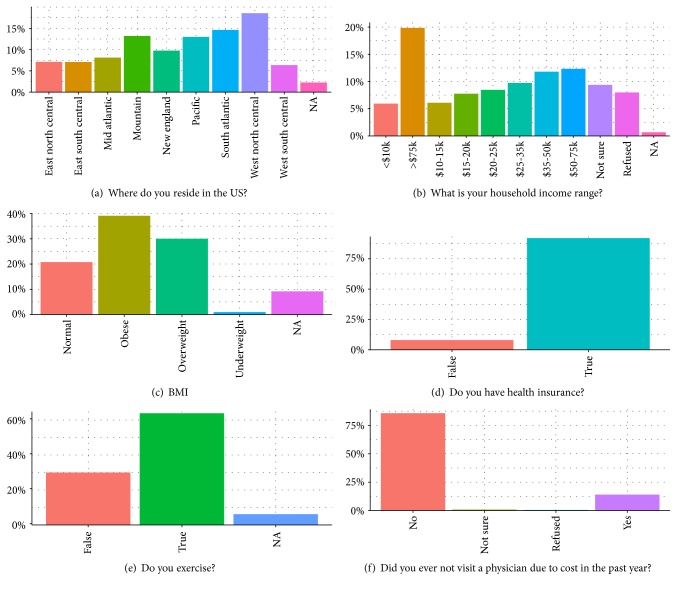
Demographics of prediabetic individuals in the United States. Source: BRFSS 2015 from http://www.Cdc.Gov.

Figure 1() depicts the demographic findings of the prediabetic individuals. Pearson's Chi-squared test on the geographic spread of the prediabetic population was found to be significant (*χ*^2^ = 170.67, *p* value < 2.2e^−16^). Of the 11,298 survey respondents, the region with the greatest percentage of prediabetic individuals (18%) was residents of the West North Central region consisting of the states of Iowa, Kansas, Minnesota, Missouri, Nebraska, North Dakota, and South Dakota. The Mountain (Arizona, Colorado, Idaho, Montana, Nevada, New Mexico, Utah, and Wyoming), Pacific (Alaska, California, Hawaii, Oregon, and Washington), and South Atlantic (Florida, Georgia, Maryland, North Carolina, South Carolina, Virginia, District of Columbia, and West Virginia) regions provided residence to over 10% of the prediabetic population. Residents in the Pacific and Mountain regions make up the Western portion of the United States, which together had 26.17% of the prediabetic population. We found that this proportion was also significant (*χ*^2^ = 51.002, *p* value = 4.888e^−11^) when we used the 4 region divisions (West, South, North, and East) similarly defined by the US Census Bureau.

Together, overweight and obese individuals comprised 69% of the prediabetic population (*χ*^2^ = 993.45, *p* value < 2.2e^−16^). Yet, over 60% of the prediabetic respondents exercise in some way, although not necessarily regularly (*χ*^2^ = 130.94, *p* value < 2.2e^−16^). Interestingly, only 19.84% of the prediabetic population had a household income greater than $75,000 per anum (*χ*^2^ = 469.48, *p* value < 2.2e^−16^). 49.77% of the respondents provided a household income range less than $50,000 per anum. 92.02% of the respondents were enrolled in a healthcare plan (*χ*^2^ = 9.3711, *p* value = 0.02474), and 14.02% refrained from visiting a physician due to medical cost (*χ*^2^ = 227.52, *p* value < 2.2e^−16^).

The findings showed that there was a correlation between household income, geographic residence in the US, and risk for developing diabetes mellitus type 2, aside from the accepted risk factors such as high BMI. A practical intervention for dealing with the increasing healthcare burden of type 2 diabetes mellitus (T2DM) must therefore be affordable and accessible to individuals of all demographics, especially those belonging to a household income less than $50,000, and be available throughout the United States, with special emphasis on the western states, while aiming to reduce the BMI of the prediabetic individuals.

## 4. Discussion

A diabetes prevention program (DPP) research study divided 3234 participants with prediabetes into three intervention groups: lifestyle modifications, metformin administration, and a control cohort using placebo [10]. The placebo cohort and the metformin cohort were given 850 mg dosage pill once daily for the first month, followed by twice daily for the remainder of the study. Lifestyle interventions were defined as diet and physical activity changes requiring at least 150 minutes of moderate intensity exercise every week, with the aim of reducing their body weight by 5–7%. The researchers monitored these prediabetic individuals over ten years, at the end of which 2531 participants remained. They assumed all the participants were enrolled in the DPP for exactly 3 years. By the 2-year mark of the study, the participants who were in the metformin group were more likely to develop diabetes than those who were in the lifestyle intervention group. Prediabetic individuals who were in the lifestyle intervention group and met the DPP criteria of losing 5–7% of their body weight decreased their risk of developing T2DM by 58%.

Consistently, for the rest of the 10-year study duration, there were always more participants in the metformin group who developed diabetes than those in the lifestyle intervention group. The placebo group had the greatest number of participants who transitioned to the diabetic state at all points of the study. This showed that lifestyle interventions and metformin administration were both good options in delaying the onset of DM in comparison to no interventions [10]. The researchers suggested that lifestyle interventions were the most cost-effective management option for prediabetes. They calculated that the direct medical costs outside of the study expenses of patients in the lifestyle intervention group equated to $26,810 over a ten-year time frame. The weighted average for the metformin group was $27,384 and for the placebo group was $29,007 [10]. The study successfully showed that from the perspective of a healthcare system, lifestyle interventions were the most beneficial for prediabetics. The authors of the study also analyzed the cost of the interventions if the patient had to pay out-of-pocket in the year 2000 for a dietician, exercise trainer, medication case manager, and so forth and determined that lifestyle interventions would still be the most cost-effective option.

A theoretical algorithm used to implement DPP on a national level in 2012 determined that by preventing 885,000 prediabetics between the ages of 18–84 from developing T2DM would tentatively save the American healthcare system $5.7 billion over 25 years [11]. However, in the initial years of implementing the program, the healthcare system would be charged $24.1 billion. It would then take thirteen years for the healthcare system to break-even from an investment point of view. Thus, lifestyle interventions would be an affordable, efficient option for implementation in prediabetic care on a national level. The at-home application of interventions would also allow individuals from all demographics to participate and incorporate changes in their lifestyles.

Unfortunately, a complex array of social, financial, behavioral, and organizational barriers impedes the application of diabetes care, especially on an individual level [12]. These multifactorial barriers can be daunting, but significant advances have occurred in learning how to translate research findings from the clinical research setting into real-world practice. To counter these barriers, implementing culturally appropriate lifestyle interventions in community settings has proven to be a prudent method for preventing diabetes.

A trial in Bronx (New York, USA) used community-engaged lifestyle coaches and academic detailers and enrolled 52 participants for a year-long, 16 sessions, diabetes prevention program based on the CDC's National Diabetes Prevention Program (NDPP). The researchers found participants had lost on average 7.4 pounds by the end of the program, lowering their risk for developing diabetes [13]. To accommodate for social discrepancies, the lifestyle coaches were trained to get along well with the members of the community they were implementing the program in.

YMCA began implementing the NDPP at their centers in collaboration with Montefiore Health Systems. Chambers et al. [14] analyzed the efficacy of the program from 14 YMCA centers in New York from 2010–2015. The participants were over the age of 18, with no previous diagnosis of diabetes, and had a BMI greater than 25 (22 if Asian), HbA1C between 5.7 and 6.4% (or the respective fasting plasma glucose or 2-hour plasma glucose range). Physicians would refer their eligible patients to the YMCA's diabetes prevention program after asking if they would like to participate. 33.6% of referred patients were enrolled for the program consisting of 16 core sessions over a one-year time frame at no cost to them. However, only 47.1% of enrollees attended at least 3 sessions. Patients who were older and fluent in English were more likely to be enrolled (defined as attending a minimum of 3 sessions) and placed in subsequent sessions. Health disparities greatly affected the practicalities of implementing the diabetes prevention program. Language and access demographics became an obstacle in program efficacy when communicating with the patients to inform them about the next session [14].

Technology may help overcome many of these disparities which prevent the NDPP from reaching its full potential. A meta-analysis of 15 published studies measuring 18 technological interventions between 2002 and 2016 analyzed the role of using technology in diabetes prevention for 2774 individuals [15]. Program lengths ranged from 12 weeks to 2 years. Interventions included DVDs, e-videos, online group forums, text messages, videoconferencing, and telephone. Collectively, these technological interventions showed an effective decrease in weight by 3.76 kilograms. Technological interventions used in collaboration with DPP programs had a greater average weight change than those with non-DPP programs. In contrast, comparison of DPP on-site versus DPP through telehealth showed that there were no significant differences in weight loss between the two types of interventions [16].

Future studies showing successful intervention of the NDPP in the United States may be a basis for application of similar interventions in other countries with high diabetic prevalence and/or economic burden. Technological interventions, their strengths and weaknesses, in the NDPP could be considered for use in countries with an ongoing diabetes control program where compliance is a problem or results are not adequate. For example, diabetes prevention in the Netherlands was addressed via lifestyle counselling and group consultations [17]. Although attendance to these sessions was high, general practitioners (GP) reported that a lack of counselling time, motivation, and financial reimbursements affected implementation success. These influencing factors may be reduced with the use of technology. Personalized phone calls providing individualized attention can compensate for the lack of counselling time, monitor patient progression, maintain motivation, and obliterate the need for financial reimbursement by reducing the number of GP visits.

The 2003 Finnish Diabetes Prevention Study showed a risk reduction of 58% after a 3.2-year intervention with diet and physical activity modifications [18]. However, the 1986 Chinese Da-Qing Study enrolled high-risk individuals with an average baseline BMI of 25.8 and found exercise alone produced the greatest risk reduction (46%) over a 6-year intervention period [18]. These findings showed that, internationally, successful implementation depended not only on effective community-based efforts, but also culturally appropriate medicine. Adapting the United States NDPP for culturally suitable applications abroad may be beneficial financially and strategically for other nations. In return, diabetes prevention programs in various countries and their respective use of cultural appropriation for increasing success rates would help the United States strategize and better adapt their DPP programs to the needs of various demographic communities domestically.

## 5. Conclusion

In conclusion, implementation of the National Diabetes Prevention Program is a rational way of reducing the burden of diabetes mellitus on the healthcare system both economically and by prevalence. However, difficulties arise in ensuring patient compliance to the program and providing access to all regions and communities of the United States. Our analysis of the BFRSS survey showed the geographic distribution of the at-risk population. Future studies analyzing the efficacy of DPP in regions outside of the East Coast United States would assist in adapting and implementing the program for all demographic populations. We also reiterated the importance of BMI and household income in this population. Lifestyle modifications, as recommended by the NDPP, aim to increase weight loss and provide a cost-effective way of reducing diabetic risk.

Major challenges remain in ensuring cultural competency of the program and program access to individuals who are busy with work or are not residing near a local center. Language and communication barriers can be accommodated with the use of technology. Technology would also help in implementing at-home changes for patients who are not able to attend on-site programs. Prediabetic individuals throughout the nation should therefore be recommended to participate in a DPP program either on-site at a local community center or remotely from home. Compliance with the program should be reinforced through the incorporation of culturally appropriated use of technology. For example, telephone monitoring and follow-ups for enrolled participants should increase their likelihood to attend the next DPP session and/or change their at-home lifestyle practices. Technology incorporation in the NDPP program would maintain a low-cost implementation by the healthcare system, be affordable and accessible for all participants, and decrease the economic burden attributed to diabetes mellitus.
